# Endogenous Pancreatic Cancer Cell PD-1 Activates MET and Induces Epithelial-Mesenchymal Transition to Promote Cancer Progression

**DOI:** 10.3390/cancers14133051

**Published:** 2022-06-21

**Authors:** Megan M. Harper, Miranda Lin, Shadi A. Qasem, Reema A. Patel, Michael J. Cavnar, Prakash K. Pandalai, Mei Gao, Joseph Kim

**Affiliations:** 1 Division of Surgical Oncology, University of Kentucky, Lexington, KY 40536, USA; megan.harper@uky.edu (M.M.H.); miranda.lin@uky.edu (M.L.); michael.cavnar@uky.edu (M.J.C.); prakash.pandalai@uky.edu (P.K.P.); mei.gao@uky.edu (M.G.); 2 Department of Pathology & Laboratory Medicine, University of Kentucky, Lexington, KY 40536, USA; s.qasem@uky.edu; 3 Division of Medical Oncology, University of Kentucky, Lexington, KY 40536, USA; reema.patel@uky.edu

**Keywords:** programmed cell death 1 receptor, proto-oncogene protein c-met, epithelial-mesenchymal transition, pancreatic neoplasm, immune checkpoint inhibitors

## Abstract

**Simple Summary:**

Here, we describe a previously unreported mechanism of PD-1/MET interaction and PD-1 induction of epithelial-to-mesenchymal transition (EMT), which is observed early in pancreatic cancer development and progression. This oncogenic mechanism is distinct from established immune functions of PD-1 and presents a new paradigm of oncogenic functionality of PD-1 in cancer cells. Our studies demonstrate the foundation and rationale for testing combination therapies targeting PD-1 and MET in pancreatic cancer patients.

**Abstract:**

We recently demonstrated that immune checkpoint PD-1 was endogenously expressed in pancreatic ductal adenocarcinoma (PDAC) cells. Our data indicated that PD-1 proteins are not exclusive to immune cells and have unrecognized signal transduction cascades intrinsic to cancer cells. Building on this paradigm shift, we sought to further characterize PD-1 expression in PDAC. We utilized a phospho-explorer array to identify pathways upregulated by PD-1 signaling. We discovered PD-1-mediated activation of the proto-oncogene MET in PDAC cells, which was dependent on hepatocyte growth factor (MET ligand) and not secondary to direct protein interaction. We then discovered that the PD-1/MET axis in PDAC cells regulated growth, migration, and invasion. Importantly, the PD-1/MET axis induced epithelial-to-mesenchymal transition (EMT), a well-established early oncogenic process in PDAC. We observed that combined targeting of PDAC cell PD-1 and MET resulted in substantial direct tumor cell cytotoxicity and growth inhibition in PDAC cell lines, patient-derived organoids, and patient-derived xenografts independent of cytotoxic immune responses. This is the first report of PDAC-endogenous PD-1 expression regulating MET signaling, which builds upon our growing body of work showing the oncogenic phenotype of PD-1 expression in PDAC cells is distinct from its immunogenic role. These results highlight a paradigm shift that the tumor-specific PD-1 axis is a novel target to effectively kill PDAC cells by antagonizing previously unrecognized PD-1-dependent oncogenic pathways.

## 1. Introduction

Pancreatic ductal adenocarcinoma (PDAC) remains a deadly disease due to a multitude of factors, including frequently late clinical presentation and a limited number of first-line therapeutic regimens that are only moderately effective [1]. Despite clinical activity in multiple cancers, immune-oncology (IO) drugs have had limited efficacy in PDAC [1]. Examining potential mechanisms of PDAC resistance to IO drugs, our group was the first to report tumor-endogenous programmed cell death protein 1 (PD-1) expression in PDAC cells [2]. Our findings challenge traditional IO dogma, which states that cancers express immune checkpoints exclusively to evade immune surveillance [3,4,5]. In this established paradigm, cancer cells express programmed death ligand 1 (PD-L1, the PD-1 ligand), which upon binding to T-cell-specific PD-1, promotes immune self-tolerance through T-cell inactivation and apoptosis [3,4,5]. However, our studies showed that PDAC cells exposed to PD-L1 activated the mitogen-activated protein kinase (MAPK) pathway. The MAPK pathway has been known to be triggered in immune cells leading to cellular energy and apoptosis [4]. However, we discovered that the PD-1/MAPK axis in PDAC cells promoted PDAC cell growth and proliferation, demonstrating a previously unrecognized PD-1-specific oncogenic phenotype that is distinct from its role in immune tolerance.

Other groups have corroborated our findings and reported other cancer cell-specific PD-1 activation of oncogenic signaling cascades [3,4,6,7,8], which appear to be dependent on the cancer histology [4,6]. Building upon our body of work showing an oncogenic role for endogenous PD-1 expression in PDAC, we discovered that highly activated c-MET (MET, mesenchymal-epithelial transition factor) was regulated by PD-1/PD-L1 signaling in PDAC cells. Classically, the tyrosine kinase receptor MET is activated when its sole ligand hepatocyte growth factor (HGF) binds, leading to receptor dimerization and tyrosine phosphorylation [9]. Although MET is normally expressed on various epithelial cells and is essential for embryonic development, organogenesis, and wound healing, MET is overexpressed and activated in 28–61% of PDACs, leading to subsequent activation of oncogenic pathways [9,10,11]. MET is also critical for intercellular signaling between epithelial and mesenchymal cells and promotes epithelial-to-mesenchymal transition (EMT), leading to decreased cell–cell adhesions and increased tumor proliferation, invasiveness, dissemination, and metastasis [9,10,11]. Based on what is known about MET, we hypothesized that tumor-endogenous PD-1 induces EMT through activation of MET to promote PDAC progression. Here, we discovered that the PDAC-endogenous PD-1/MET axis increased growth, migration, and invasion. These outcomes appear to be dependent on HGF, the ligand of MET, rather than direct protein interactions between PD-1 and/or PD-L1 and MET. Importantly, we also discovered that the PD-1/MET axis induced EMT. Altogether, our studies revealed a novel and immune-independent mechanism for tumor-intrinsic PD-1/MET to enhance an invasive PDAC phenotype.

Since our studies revealed potential PDAC dependence of PD-1/MET oncogenic signaling, we sought to determine whether therapeutic targeting of this axis could block tumor growth. In order to minimize the time from bench-to-bedside and promote rapid development of new clinical trials and improve survival of this deadly disease, we preferentially utilized current clinical drugs to investigate this unexplored PD-1/MET mechanism in PDAC. Our assays revealed that direct targeting of the PD-1/MET axis with a combination of drugs resulted in synergistic and direct PDAC cell cytotoxicity and inhibited tumor growth independent of the immune system. These results have important implications suggesting that disruption of PD-1-specific oncogenic dependence in PDAC may lead to effective killing of PDAC cells. Our findings establish the basis for combined anti-PD-1/anti-MET therapies in PDAC and provide a promising novel therapeutic approach to improve survival for current PDAC patients with this devastating disease.

## 2. Materials and Methods

Additional details are listed in Supplementary File S1.

### 2.1. Drugs

Anti-PD-1 monoclonal antibody (mAb) pembrolizumab (PEM), cabozantinib (CABO, small molecule anti-MET, -VEGFR2, -AXL, and -RET), and tivantinib (TIV, small molecule anti-MET) were utilized given their current clinical indications. Additional information is listed in Table S1.

### 2.2. Cell Culture

MIAPaCa-2 and PANC-1 cells were obtained from and validated by American Type Culture Collection (ATCC). Cells were maintained as described [2]. Testing for *Mycoplasma* contamination, which can cause chromosomal mutations and genetic drift from parental cell lines, is recommended after 10 passages to ensure. All experiments utilized early passage (< P10) cells. However, given the time between receiving the P0 cells from ATCC, cell banking, and subsequent use of early passage cells, we validated the genomic integrity of our cell lines through ATCC. Validation of genomic integrity mitigates the concern for *mycoplasma* contamination. *PD-1* knockdown (KD) PDAC cells were generated as reported [2].

### 2.3. Ethics Approval and Patient Tumor Acquisition

The University of Kentucky (UK) Institutional Review Board approval and written informed patient consent were obtained prior to obtaining tissue samples in accordance with the Declaration of Helsinki. Excess cancer tissues were processed to create patient-derived organoids (PDOs) and xenografts (PDXs) as described [2,12,13].

### 2.4. Western Blots

Cell and PDO lysates were collected and analyzed by western blot as described [2,13]. For EMT studies, cells were grown to 60–80% confluence to capture mesenchymal processes. Cells were serum-starved overnight prior to treatment with PD-L1 (1 µg/mL), vehicle-control (0.1% BSA), or when evaluating EMT and phosphorylated proteins. When noted, cells were treated with PEM (250 µg/mL) or TIV (0.25 µM) 30 min before PD-L1 stimulation. Blots were probed independently with antibodies listed in Table S1. Blots were visualized with ECL solution. Quantified expression values were normalized to controls from the same blot and from at least two separate experiments. Original western blot images can be found in Supplemental File S2.

### 2.5. Co-Immunoprecipitation

Co-immunoprecipitation (co-IP) assays were performed per the manufacturer’s instructions. One milligram of pre-cleared lysates was incubated overnight at 4 °C with 75 μg polyclonal anti-PD-1, anti-PD-L1, or IgG antibodies.

### 2.6. Quantitative PCR

Cells were treated with recombinant-human PD-L1 (1 µg/mL) or solvent-control (0.1% BSA) for 2 h. Cells were lysed, total RNA extracted, and reverse transcription performed per the manufacturers’ instructions. Quantitative real-time PCR (qRT-PCR) was performed in quadruplicate using TaqMan reagents per the manufacturer’s instructions. Data were analyzed using the 2^−***ΔΔCT***^ method with results normalized to β-actin.

### 2.7. Wound Healing

Due to the different sizes of the PDAC cells utilized, 7.5 × 10^5^ cells/well of MIAPaCa-2 cells and 5 × 10^5^ cells/well of PANC-1 cells were initially plated in 6-well plates. Scratches were made in confluent cell monolayers on the 2nd day after plating. Cells were treated in triplicate with solvent-control (0.1% BSA), TIV (0.25 µM), PEM (250 µg/mL), recombinant-human HGF (100 ng/mL; positive-control), PD-L1 (1.5 µg/mL), PD-L1 + TIV, or PD-L1 + PEM. Three representative images were taken immediately after and 24 h after treatment.

### 2.8. Transwell Cell Migration

Upper chambers of 24-well transwell inserts (8 μm pores, Costar) contained 5 × 10^4^ PDAC cells resuspended in 100 μL serum-free media. Bottom chambers included 600 μL of medium + 2% FBS with the above treatments. MIAPaCa-2 and PANC-1 cells were incubated for 12 h and 18 h, respectively [14]. Cells were fixed and permeabilized in ice-cold 100% methanol and stained with 1% crystal violet solution. The stained transwell membranes were imaged with a Nikon Ts2 microscope. Cell densities were analyzed using 3 representative images for each well with ImageJ software (v1.53) and graphed with GraphPad Prism (v9).

### 2.9. Drug Cytotoxicity Assays

Cytotoxicity assays were performed as described [2,12,13]. Cells and PDOs were seeded in 96-well plates at 4 × 10^3^ cells/well and 2 × 10^3^ cells/10 μL BME/well, respectively. Results are representative of three experiments in triplicate. Combination treatments were performed using the lowest IC_50_ values for each drug. Cells were treated with IgG4 (PEM negative-control), solvent (CABO/TIV negative-control), CABO, TIV, PEM (1 mg/mL), CABO + PEM, or TIV + PEM for 48 h.

### 2.10. In Vivo Drug Testing

The UK Institutional Animal Care and Use Committee approved the described murine studies. ARRIVE guidelines were followed (Supplementary File S1, Table S2). PDX mice were randomly divided into the following treatment groups (N = 6 per group) and treated for four weeks: control, TIV (150 mg/kg, oral gavage every 5 d + 2 d holiday), PEM (30 mg/kg, intraperitoneal injection twice weekly), TIV + PEM [15]. Researchers were not blinded to treatment groups.

### 2.11. Immunohistochemical (IHC) Staining

Excised PDX tumors were fixed in 10% formalin and submitted to the UK Biospecimen Procurement and Translational Pathology (BPTP) Shared Resource Facility (SRF), which performed all histological sectioning and staining (Supplementary File S1).

### 2.12. Statistical Analysis

Experiments were performed at least twice. Statistical analyses were performed using GraphPad Prism software (v9). For two-group analysis, two-tailed Student’s *t*-tests were used. One- and two-way ANOVA with post-hoc Tukey tests were used for multi-group comparisons. Differences were considered significant at *p* < 0.05. Results were graphed as mean ± standard error. For multi-treatment studies, percent and fold-changes were calculated compared to controls to enable easier comparison across all data.

## 3. Results

### 3.1. Identification of PD-1/PD-L1 Activated Pathways

We identified proteins and signaling pathways enhanced by PD-1/PD-L1 signaling. After PANC-1 cells were exposed to PD-L1, our phospho-protein array demonstrated upregulated expression changes (fold-change > 2) in histone deacetylase (HDAC)-1 (28.98-fold), MET (13.65-fold) [16], and EGFR (6.4-fold) (Figure S1). Since anti-HDAC therapies are non-selective and target epigenetic alterations [17] and anti-EGFR therapies such as erlotinib have failed to show clinically meaningful benefit in PDAC [18,19], we chose to investigate MET, which is a promising therapeutic target for various cancers and is overexpressed, upregulated, and inversely associated with prognosis in PDAC [9,10,11]. Interestingly, MET therapies have been shown to overcome anti-EGFR monotherapy and gemcitabine resistance in non-small cell lung cancer (NSCLC) and PDAC [9]. Furthermore, MET regulates EMT, tumor growth, metastases, and interacts with numerous oncogenic pathways in PDAC [9]. These features provided a strong rationale to focus on MET for our studies.

### 3.2. PD-1 and MET Expression in PDAC Cells and PDOs

**Figure 1:**
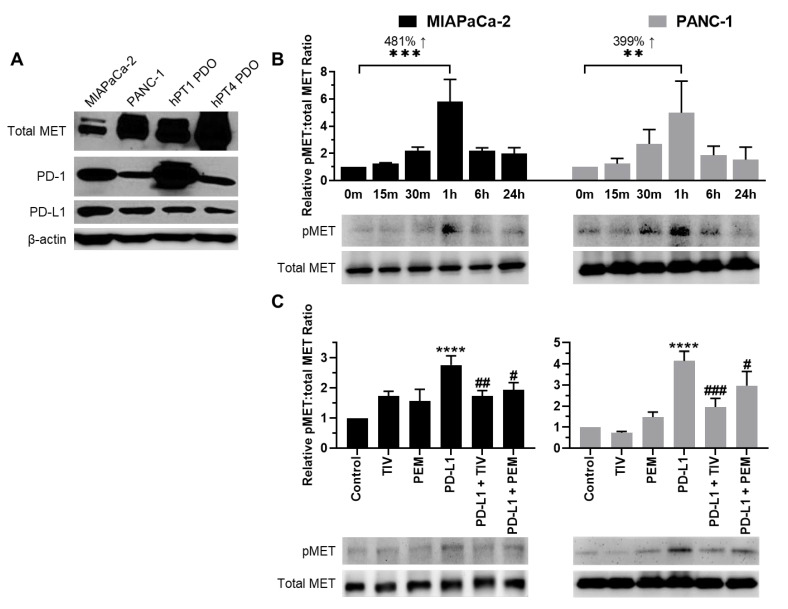
**PD-1/PD-L1 axis induces MET activation.** (**A**) Innate PD-1, PD-L1, and total MET expression in PDAC cells and PDOs. Β-actin was used as an internal control. (**B**) PD-L1-induced MET activation (pMET) was time-dependent and peaked after 1 h of treatment, increasing 481% for MIAPaCa-2 and 399% for PANC-1 cells. pMET levels were compared to total MET (**C**) TIV and PEM both blocked PD-L1-induced MET activation at 1 h in PDAC cells. Total MET was used as a loading control. ** *p* < 0.01, *** *p* < 0.001 vs. 0 min; **** *p* < 0.0001 vs. control; # *p* < 0.05, ## *p* < 0.01, ### *p* < 0.001 vs. PD-L1.

We detected the expression of PD-1, PD-L1, and MET in PDAC cells and PDO lines by western blot assays. Consistent with the Cancer Cell Line Encyclopedia (CCLE) data [20], our results showed that MIAPaCa-2 and PANC-1 cell lines expressed all three proteins (Figure 1A). However, the highest levels of PD-1 and total-MET expression were observed in PDO lines hPT1 and hPT4, respectively. Following exposure to recombinant-human PD-L1, MIAPaCa-2 and PANC-1 cells both demonstrated time-dependent alterations in MET activation, which peaked at 1 h with 481% and 399% increases, respectively, in phosphorylated-MET (pMET) compared to baseline (*p* < 0.01 vs. 0 min for all) (Figure 1B). In blocking assays, PD-L1 treatment alone resulted in 177% and 314% increased pMET levels for MIAPaCa-2 and PANC-1, respectively. Pretreatment with PEM and TIV abrogated PD-L1-induced pMET levels by 83% and 103%, respectively, for MIA-PaCa-2 and 117% and 219%, respectively, for PANC-1 (Figure 1C). These results demonstrated that PD-L1-induced MET activation is dependent on both MET and PD-1.

### 3.3. PD-1 and MET Regulate Cell Motility and Migration

**Figure 2:**
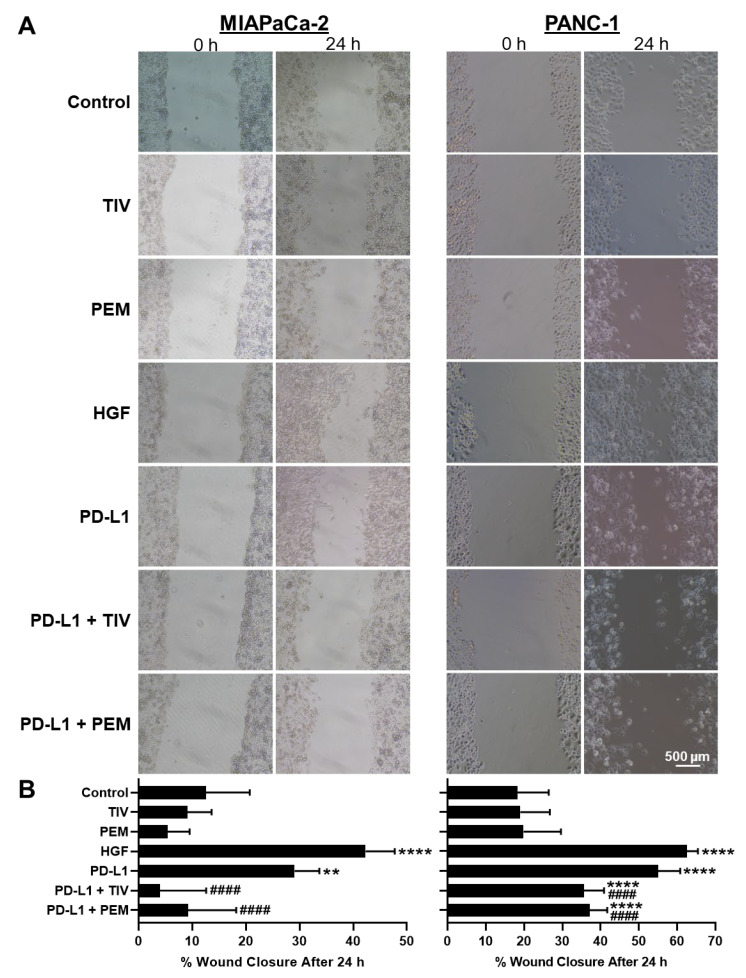

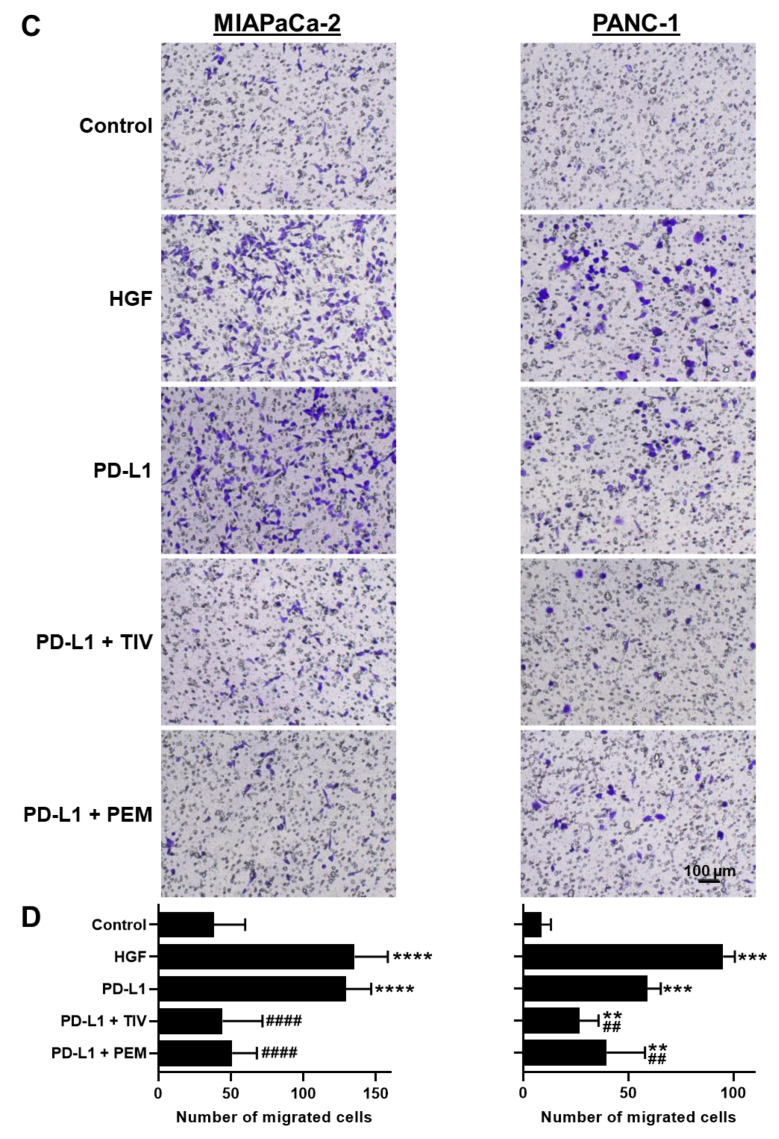
**MET inhibition can block PD-L1-induced cell motility and migration.** (**A**) Representative images of scratch assays after 24 h in PDAC cells. (**B**) Quantification showing the percent of wound closure after 24 h. (**C**) Representative images and (**D**) quantification of transwell migration assays. ** *p* < 0.01, *** *p* < 0.001, **** *p* < 0.0001 vs. control, ## *p* < 0.01, #### *p* < 0.0001 vs. PD-L1.

Given that MET induces EMT, we sought to determine if the PD-1/PD-L1-axis regulates this process to promote motility and migration. To assess cell motility, we performed wound healing assays in PDAC cells. We observed that PD-L1 treatment significantly increased motility in MIAPaCa-2 and PANC-1 cells compared to negative-controls (132% and 201%, respectively; *p* < 0.01) (Figure 2A,B). These motility increases were consistent with HGF positive-control results (237% and 242% for MIAPaCa-2 and PANC-1, respectively), demonstrating that the PD-1/PD-L1-axis induces PDAC cell motility similar to direct MET activation. Additionally, both PD-1 and MET antagonism completely blocked PD-L1-induced cell motility in MIAPaCa-2 cells. In PANC-1 cells, compared to negative-control, PD-L1-induced cell motility decreased by 106% and 98% following PD-1 or MET antagonism, respectively (all *p* < 0.0001 vs. control). This further supported that PD-L1-induced cell motility is dependent on both PD-1 and MET.

To evaluate PD-1/PD-L1/MET activity on cell migration, we next performed transwell migration assays. In both MIAPaCa-2 and PANC-1 cells, PD-L1 treatment alone increased cell migration (138% and 470%, respectively, vs. negative-control). These results were comparable to the increased migration observed with HGF positive-controls (153% and 881% for MIAPaCa-2 and PANC-1, respectively, vs. negative-control). Conversely, compared to the negative control, when PD-L1 treatment was combined with PD-1 or MET inhibition, cell migration was nearly completely blocked for MIAPaCa-2, and decreased by 366% and 220%, respectively, for PANC-1 (all *p* < 0.01 vs. no treatment) (Figure 2C,D). The above assays showed that PD-L1-induced motility/growth and migration are both dependent on PD-1 and MET.

### 3.4. The PD-1/PD-L1 Axis Induces EMT

**Figure 3:**
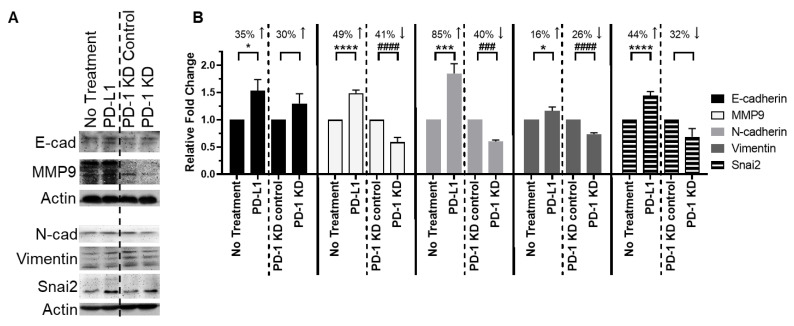
**PD-1/PD-L1 axis controls EMT in PDAC cells.** (**A**) Representative blots and (**B**) quantification of EMT expression patterns in PANC-1 cells. PD-L1 treatment increased mesenchymal markers MMP9, vimentin, and Snai2 expression. *PD-1* knockdown cells showed E-cadherin/N-cadherin expression pattern switching and decreased mesenchymal-associated protein expression (MMP9, vimentin, and Snai2). PD-L1 treatment is compared against no treatment. *PD-1* KD is compared against *PD-1* KD control. Dashed vertical lines are used to distinguish the comparison arms for clarity. * *p* < 0.05, *** *p* < 0.001, **** *p* < 0.0001 vs. no treatment; ### *p* < 0.001, #### *p* < 0.0001 vs. KD control.

We hypothesized that the PD-1/PD-L1 axis regulates EMT. We used PANC-1 cells for these assays, given their epithelial phenotype compared to the baseline mesenchymal phenotype of MIAPaCa-2 cells. After 48 h of PD-L1 stimulation, PANC-1 cells demonstrated EMT features with increased expression of mesenchymal markers MMP9 (49%), N-cadherin (85%), vimentin (16%), and Snai2 (44%) compared to untreated controls (Figure 3). E-cadherin increased following treatment with PD-L1 (35%), which is likely due to the increased formation of cell-to-cell adhesions in the interior of cell colonies observed during rapid cell growth [21,22,23]. These interior cells appear to express EMT factors that are unrelated to cell-to-cell interactions. In comparison, N-cadherin is expressed only on cells along the periphery of colonies without cell-to-cell interactions, thus representing the minority of cells.

To corroborate that PD-L1 stimulation increased EMT marker expression through PD-1, we utilized *PD-1* KD cells. With *PD-1* knockdown, we observed E-cadherin/N-cadherin pattern switching, where E-cadherin increased 30% and N-cadherin decreased 40% vs. untreated controls. Furthermore, mesenchymal-related proteins MMP9, vimentin, and Snai2 decreased in *PD-1* KD cells (41%, 26%, and 32%, respectively). These results demonstrate that induction of mesenchymal protein expression is dependent on PD-1, supporting our results showing that PD-L1-induced migration and motility are dependent on PD-1. Taken together, these data demonstrate a novel mechanism for the regulation of EMT by the PD-1/PD-L1 axis.

### 3.5. PD-1 and PD-L1 Do Not Directly Interact with MET

We next sought to investigate if the mechanism for PD-L1-induced MET activation involved a direct PD-1–MET protein interaction. We performed co-IP assays utilizing PD-1 as the “bait” in PDAC cells. Co-IP protein complex samples were analyzed by western blot with anti-MET and anti-PD-1 mAbs (“prey”). We observed no difference in MET nor PD-1 expression between IgG control and co-IP samples (Figure S2A), indicating there is likely no direct protein interaction between MET and PD-1 in PDAC cells.

To evaluate if PD-L1-induced MET activation is due to exogenous PD-L1 directly binding to and stimulating MET, we performed additional co-IP assays with PD-L1 as the “bait.” Cells were treated with PD-L1 for 1 h prior to lysate collection. PD-L1 pull-down did not show increased levels of MET compared to IgG controls, demonstrating there is likely no direct PD-L1–MET interaction and PD-L1 likely does not act as a ligand for MET (Figure S2B). These findings suggest an alternative, indirect pathway for PD-1/PD-L1-induced MET activation.

### 3.6. PD-1/PD-L1 Axis Upregulates HGF mRNA Expression

**Figure 4:**
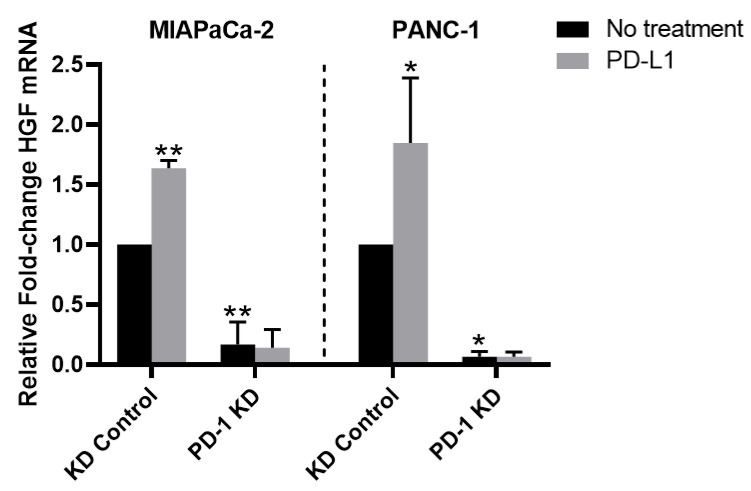
**PD-L1 increases HGF mRNA expression.** HGF mRNA expression in MIAPaCa-2 and PANC-1 *PD-1* KD control cells increased by 64% and 84%, respectively, following PD-L1 treatment. Knockdown of *PD-1* decreased baseline HGF mRNA expression by 499% and 1532% compared to KD controls, respectively. Treatment with PD-L1 was unable to overcome this reduction in HGF in *PD-1* KD cells, signaling PD-1 is required for PD-L1-induced HGF mRNA production. * *p* < 0.05, ** *p* < 0.01 vs. untreated KD controls.

We hypothesized that PD-1/PD-L1 indirectly activates MET through HGF expression. We assessed *HGF* mRNA expression following PD-L1 stimulation in *PD-1* KD PDAC cells by qPCR (Figure 4). Following exposure to PD-L1, MIAPaCa-2 and PANC-1 *PD-1* control cells demonstrated 64% and 84% increased *HGF* mRNA, respectively (*p* < 0.05 vs. KD control for all). To determine if PD-1 is a requisite for PD-L1-mediated HGF expression, we evaluated *HGF* mRNA levels in *PD-1* KD cells. We detected minimal expression of *HGF* mRNA in *PD-1* KD cells compared to control cells. Furthermore, PD-L1 exposure in *PD-1* KD cells did not alter HGF expression levels, indicating that HGF expression is dependent on both PD-1 and PD-L1. These findings support our earlier data showing that PD-L1-induced MET activation is dependent on PD-1.

### 3.7. PD-1 and MET Inhibitors Have Synergistic Cytotoxicity in PDAC Cells and PDOs

**Figure 5:**
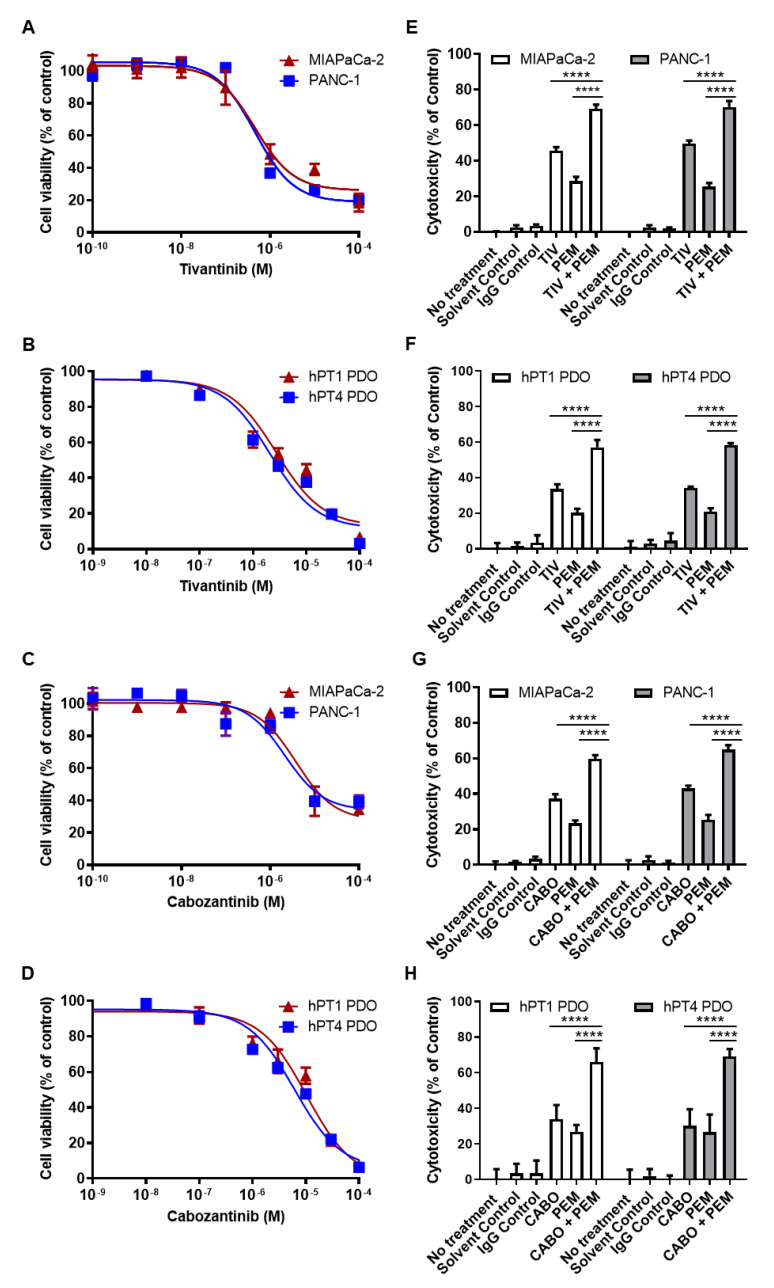
**TIV and CABO demonstrate synergistic cytotoxicity with ICIs in PDAC Cells and PDOs**. Dose–response curves of PD-1 (+), MET (+) PDAC cells and PDOs after treatment with different concentrations of TIV (**A**,**B**) or CABO (**C**,**D**). Combination cytotoxicity assays with TIV + PEM (**E**,**F**) or CABO + PEM (**G**,**H**) demonstrated synergistic cytotoxicity compared to control and single-drug treatment arms and produced the greatest cytotoxicity in all lines. **** *p* < 0.0001 vs. TIV, CABO, or PEM.

Next, we examined the direct cytotoxic effects of anti-PD-1 and anti-MET drugs in vitro. We treated MIAPaCa-2 and PANC-1 cells with the MET inhibitors TIV and CABO and calculated IC_50_ for each cell line (TIV: 0.487 and 0.456 µM, respectively; CABO: 3.78 and 2.12 µM, respectively) (Figure 5). For subsequent assays, we used the lowest IC_50_ concentration for each drug (TIV: 0.5 µM, CABO: 2 µM). We then performed single and combination drug sensitivity testing and found the highest cytotoxicity with combined TIV + PEM (MIAPaCa-2: 69% vs. TIV 46%, PEM 29%; PANC-1: 70% vs. TIV 50%, PEM 25%) and CABO + PEM (MIAPaCa-2: 60% vs. CABO 37%, PEM 23%; PANC-1: 65% vs. CABO 43%, PEM 25%) (*p* < 0.0001).

In order to evaluate the potential synergistic antagonism of PD-1 and MET in a more clinically relevant model with a tumor microenvironment mirroring the original tumor [24,25,26,27,28], we next performed drug testing in PD-1^+^/MET^+^ PDAC PDOs hPT1 and hPT4. IC_50_ values for TIV and CABO were determined for each line (TIV: 2.75 and 2.09 µM, respectively; CABO: 10.4 and 6.15 µM, respectively) (Figure 5). We utilized the lowest IC_50_ concentration for each drug from PDAC cell and PDO testing (TIV: 0.5 µM, CABO: 2 µM) for subsequent drug sensitivity testing. As in PDAC cells, PDOs demonstrated the highest cytotoxicity with combined TIV + PEM (hPT1: 57% vs. TIV 34%, PEM 20%; hPT4: 58% vs. TIV 34%, PEM 21%) and CABO + PEM (hPT1: 66% vs. CABO 34%, PEM 27%; hPT4: 69% vs. CABO 30%, PEM 27%) (*p* < 0.0001). Altogether, our in vitro drug testing results demonstrate that combined PD-1/MET antagonism enhances direct tumor cytotoxicity compared to monotherapy regimens.

### 3.8. PD-1 and MET Inhibition Effectively Slows Tumor Growth in PDXs

**Figure 6:**
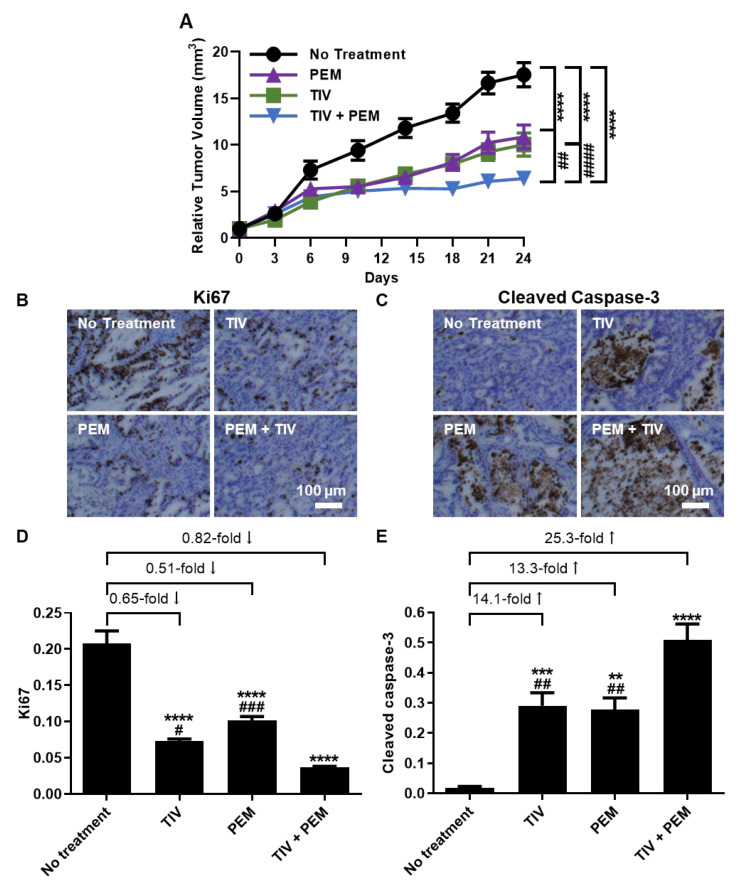
**Combined PD-1 and MET antagonism acts synergistically in vivo to decrease tumor burden, decrease proliferation, and increase direct tumor cytotoxicity.** (**A**) Relative tumor volume of each group revealed the slowest tumor growth occurred in mice treated with TIV + PEM. IHC staining (**B**,**C**) and quantification (**D**,**E**) of PDX tumors using proliferation marker ki67 (**B**,**D**) and cell apoptosis marker cleaved caspase-3 (**D**,**E**). ** *p <* 0.01, *** *p <* 0.001, **** *p <* 0.0001 vs. control; # *p <* 0.05, ## *p <* 0.01, ### *p <* 0.001, #### *p <* 0.0001 vs. TIV + PEM.

We next sought to test our mechanism-based regimen in vivo. We established PDX tumors generated from hPT1. PDX mice were given vehicle-control, TIV, PEM, or TIV + PEM as described earlier. We utilized TIV for these studies given its selective MET antagonism, thereby enabling evaluation of the in vivo PD-1/MET axis mechanism without the influence of off-target effects. We observed the slowest tumor growth with combined TIV + PEM treatment (Figure 6A). Furthermore, IHC staining of cell proliferation marker protein Ki67 (Figure 6B) and cell apoptosis marker cleaved caspase-3 (Figure 6C) showed that TIV and PEM monotherapy cohorts inhibited tumor cell proliferation (decreased 0.65-fold and 0.51-fold vs. control, respectively) and promoted tumor cell apoptosis (increased 14.1-fold and 13.3-fold vs. control, respectively). However, this effect was greatest with combined PD-1/MET antagonism (decreased proliferation 0.82% and increased apoptosis 25.3-fold vs. control) (Figure 6D,E, all *p* < 0.05). These data demonstrate in vivo synergistic efficacy with combined PD-1/MET antagonism that inhibits tumor growth, decreases proliferation, and promotes direct tumor cytotoxicity. Importantly, our in vivo studies show direct tumor cell targeting, similar to that observed in our in vitro results, in the presence of a complex tumor microenvironment. Furthermore, these results demonstrate a novel mechanism of action for anti-cancer IO therapies apart from immune reactivation.

## 4. Discussion

Our current studies demonstrated endogenous PD-1 expression in PDAC cells and tissues and revealed that PD-1 signaling upregulated oncogenic MET activity to subsequently induce EMT. The dependence of PDAC growth on the PD-1/MET axis was substantiated by high levels of cytotoxicity when both PD-1 and MET were therapeutically targeted. These data, along with our prior report [2], continue to challenge the paradigm that PD-1 expression is exclusive to T-cells and cytotoxic immune responses. Instead, our results involving the PD-1/MET axis in PDAC cells underscore the growing body of work showing that the tumor-endogenous immune checkpoint PD-1 has oncogenic properties supporting cancer progression. Examples of such dual functionality of proteins are evident even from our prior studies on chemokine receptors [29,30,31]. G-protein coupled receptor expression in immune cells has physiologic roles in organogenesis, development, and inflammation [32]. In cancer cells, however, chemokine receptors (e.g., CXCR4) have been shown to support an invasive phenotype [29,30,31,33].

PD-1 regulation of MET, which is an important proto-oncogene for many cancers, underlies how this axis supports PDAC progression. MET is well-known to activate numerous downstream oncogenic pathways, induce EMT signals, and promote cancer growth, invasion and metastases [9,10]. In fact, MET overexpression has been detected in nearly two-thirds of PDACs and has been associated with worse clinical outcomes [11]. Interestingly, other recent studies have shown that activated MET and EMT pathways increase tumor-endogenous PD-L1 production [34,35]. These studies, along with our results, identify a potential positive feedback loop and will be explored in future investigations. Nevertheless, in this report, we are the first to demonstrate that tumor-endogenous PD-1 promotes oncogenic MET signaling to induce EMT and promote PDAC progression.

Given that our data demonstrated that the PD-1/MET axis supported PDAC growth, we sought to test whether therapeutic targeting would substantiate PDAC growth dependency on this pathway. Indeed, our results revealed that combined anti-PD-1/-MET antagonism resulted in high levels of PDAC cytotoxicity in both in vitro and in vivo cancer models. There are important implications from these results. First, our assays revealed that single-agent regimens were suboptimal and that combination regimens yielded higher synergistic cytotoxic responses. These results may suggest that alternate or escape signaling pathways are not attenuated with single-agent regimens. Second, our drug treatment assays were performed in immune-deficient PDAC models, and the high levels of cytotoxicity challenge the concept that IO anti-cancer efficacy is derived only from immune reactivation. Notwithstanding, it is reasonable to deduce that in the clinical management of PDAC patients, therapeutic regimens that incorporate IO drugs combined with targeted therapies may produce optimal cytotoxicity from both harnessing adaptive immune responses as well as from direct cytotoxic killing. Our group is developing immune-competent PDAC models to examine both avenues of cytotoxicity in our future studies.

Our study results are of particular clinical importance given that there has been slow progress on the development of novel effective therapeutic regimens for metastatic PDAC since FDA approval of first-line regimens FOLFIRINOX and Gemcitabine/nab-Paclitaxel in 2010 and 2013, respectively, and second-line regimen NALFIRIFOX in 2015 [36,37,38]. Regimens that provide durable improvements in the long-term survival of this disease are lacking, creating an urgent need for innovative therapeutics. The CheckMate 9ER trial was a landmark study combining an IO drug (nivolumab, anti-PD-1 mAb) and multi-kinase inhibitor with anti-MET properties (CABO) and has resulted in a new standard-of-care first-line therapy for advanced renal cell carcinoma (RCC) [39]. Trials such as CheckMate 9ER provide foundational support to investigate combined anti-PD-1/anti-MET therapies in PDAC patients. Consequently, we have organized a clinical trial evaluating CABO + PEM in advanced PDAC (NCT05052723). This is the first clinical evaluation of combined PD-1/MET antagonism in PDAC.

## 5. Conclusions

Our data challenges the current paradigm of immune checkpoints and immune tolerance by showing that tumor-intrinsic PD-1 supports an oncogenic phenotype in PDAC apart from its role in the inhibition of cytotoxic immune responses. Our studies revealed a novel mechanism wherein tumor-endogenous PD-1 increased MET activation and induced EMT in PDAC. This oncogenic signaling axis was targeted to effectively promote direct tumor cytotoxicity in PDAC cells and tissues. These study results served as the basis for a current clinical trial testing the combination of PEM and CABO in metastatic PDAC (NCT05052723).

## Data Availability

Data presented within the scope of this article are available upon request from the corresponding author.

## Supplementary Materials

The following supporting information can be downloaded at: https://www.mdpi.com/article/10.3390/cancers14133051/s1, Figure S1: Phospho-protein array results. Figure S2: MET does not directly interact with PD-1 or PD-L1. Table S1: Drugs and reagents. Table S2: Descriptive statistics of PDX studies. File S1: Supplemental materials & methods. File S2: Original western blot images.
