# Supplementary material for: Endogenous Pancreatic Cancer Cell PD-1 Activates MET and Induces Epithelial-Mesenchymal Transition to Promote Cancer Progression
Source: Cancers (Basel). 2022 Jun 21;14(13):3051. doi: 10.3390/cancers14133051 (PMC9264908; doi:10.3390/cancers14133051)
Supplement: Supplementary file 1 [file cancers-14-03051-s001.zip › Supplementary File S1.pdf]

SUPPLEMENTARY MATERIALS AND METHODS

Table S1: Drugs and Reagents

#	Drugs/Treatments	Target/ Reagent Name	Company	Catalog Number
1	Cabozantinib	anti-MET, -VEGFR2, -AXL, & -RET	LC Labs	8999
2	Pembolizumab	Anti-PD-1	Merck	NDC 0006-3026-01
3	Tivantinib	Anti-MET	MedChemExpress	HY-50686
4	Recombinant human HGF	MET	SinoBiologic	10463-HNAS
5	Recombinant human PD-L1	PD-1	Peprotech	310-35
6	**Drug Solvents**	BSA	Sigma	A7030
7		20% Vitamin E TPGS	Sigma	57668
8		Normal Saline (0.9% NaCl)	Hospira	NDC 0409-1966-02
9		PEG 400	Baker	U216-01
10	**Antibodies**	β-actin (mAb)	Sigma	A1978
11		Ki67	Abcam	ab16667
12		Cleaved-caspase 3	Cell Signaling Technology (CST)	9661
13		E-cadherin	CST	3195
14		MET	CST	8198
15		p-MET	CST	3077
16		MMP9	Proteintech	10375-2-AP
17		N-cadherin	Proteintech	22018-1-AP
18		PD-1 (mAb)	Proteintech	66220-1-Ig
19		PD-1 (polyclonal)	Proteintech	18106-1-AP
20		PD-L1 (mAb)	Proteintech	66248-1-Ig
21		PD-L1 (polyclonal)	Proteintech	17952-1-AP
22		Snai2	CST	9585
23		Vimentin	CST	5741
24		IgG4κ	Novusbio	DDXCH041-100
25		Polyclonal IgG	Proteintech	30000-0-AP
26	**shRNA KD Reagents**	Anti-*PD-1* shRNA lentiviral vector	Genecopedia	LPP-HSH012662-LVRU6MP
27		Control scramble shRNA lentiviral vector	Genecopedia	LPP-CSHCTR001-LVRU6MP
28		jetPRIME reagent	Polyplus	101000015

29		pMD2.G envelope plasmid	Addgene	12259
30		psPAX2 packaging	Addgene	12260
31	**Other Reagents**	BME	R&D	3533-005-02
32		CellTiter Glo	Promega	G9242
33		high-capacity RNA-to-cDNA Kit	Applied Biosystems	4387406
34		Phospho Explorer Antibody Array	Full Moon Biosystems	KAS02
35		Pierce™ Co-Immunoprecipitation Kit	Thermo	26149
36		Puromycin	Thermo	A1113803
37		RiboPure™ Kit	Invitrogen	AM1924
38		SuperSignal West Pico Chemiluminescent Substrate	Thermo	34577
39		TaqMan ACTB probes	Applied Biosystems	Hs99999905_m1
40		TaqMan HGF probes	Applied Biosystems	Hs00300159_m1
41		TaqMan Master Mix	Applied Biosystems	4440038
42		Transwell cell culture plates	Costar	3464
43		TRI Reagent Solution	Invitrogen	9738
44		WST-1 Kit	TaKaRa	MK400

Phospho-protein array

Based on RNAseq data from Cancer Cell Line Encyclopedia (CCLE) [20], PANC-1 cells express the highest levels of PD-1 compared to other PDAC cell lines and were utilized for this assay. After exposure to recombinant-human PD-L1 (1 µg/ml) for 15 min, changes in phospho-proteins were assessed in cell lysates using the Phospho Explorer Antibody Array, an ELISA based assay that measures expression levels of >200 phosphorylated and corresponding total proteins, as previously described [33]. The manufacturer performed all array analyses.

Patient derived organoids (PDOs)

PDOs were created as previously described [2,12,13] In brief, tumor tissues were mechanically minced and then digested in collagenase, dispase, and DNase and incubated at 37°C for 30 min with intermittent agitation. Advanced DMEM/F12 wash medium was added to neutralize digestion.

Dissociated tumor material was collected and suspended in Cultrex® Reduced Growth Factor Basement Membrane Matrix, Type 2 (RGF BME-2), plated on pre-warmed 24-well plates, and overlaid with complete organoid media containing growth factors. Complete PDO media was changed every other day. PDOs were passaged as previously described at 80-90% confluence.

Quantitative PCR

Reverse transcription was performed on a C1000 Touch Thermal Cycler (Bio-Rad). The cycling conditions for cDNA generation were set at 37°C for 60 min, 95°C for 5 min, followed by a 4°C hold per the manufacturers' recommendations. qRT-PCR was then carried out on a QuantStudio 3 Real-Time PCR System (Thermo) using the following cycling conditions: 50°C for 2 min, 95°C for 10 min, 40 cycles of 95°C for 15 s and 60°C for 1 min.

***PD-1* knockdown in PDAC cells**

Lentiviral short-hairpin RNA (shRNA) against human *PD-1* (*PDCD1*) (NM_005018), or control scramble shRNA plasmids, with the mCherry reporter and puromycin resistance genes were obtained from Genecopoeia as reported previously [2]. Plasmids were amplified and transfected into MIAPaCa-2 and PANC-1 cells using the jetPRIME reagent. Successfully transfected cells were selected with puromycin added to cell culture media. Following selection, cells were further purified for high plasmid expression by flow cytometry using the mCherry reporter. The cells expressing the highest levels of mCherry (top 10-50% of total selected cells) were isolated for further use. *PD-1* knockdown efficiency was assessed by western blot and the most efficient *PD-1* and control shRNA vectors were packaged with psPAX2 packaging into the pMD2.G envelope plasmid at a ratio of 5:3:2 and transfected into HEK293T cells validated by American Type Culture Collection for packaging. Viral particles were harvested after 48 h and used for transduction in PDAC cells. Infected PDAC cells were further flow-sorted for high mCherry expression as noted.

Western Blots

Primary antibodies against PD-1 (1:500), PD-L1 (1:1000), N-cadherin, (1:500), MET (1:1000), phospho-MET (1:1000), E-cadherin (1:500), Snai2 (1:500), vimentin (1:2000), MMP9 (1:1000) were used. Where shown, total MET and β-actin (1:5000) were used as internal loading controls. Blots were visualized using an UVP ChemiDoc-It2imager and/or standard film. Western blot data were quantified by ImageJ (NIH) and Image Studio (LiCor) software.

Drug cytotoxicity assays

MIAPaCa-2 and PANC-1 cells and PDOs were seeded in 96-well plates at $4x10^3$ cells/well and $2x10^3$ cells/10 μl BME/well, respectively. PDAC cells and PDOs and were exposed to CABO or TIV for 48 h at 8 concentrations ranging between 0-100 μM on days 1 or 3, respectively. Cell viability was measured using a WST-1 assay kit for PDAC cells and CellTiter for PDOs. Absorbance was read on a Synergy HTX Multi-Mode Reader (BioTek). Dose-response curves were generated and the IC_{50} values were calculated for each cell line with GraphPad Prism software.

***In vivo* drug testing**

Female Nod-Scid-γ (NSG) mice (5-8 weeks old, The Jackson Laboratory) were acclimated to the animal housing facility for at least 1 week prior to study initiation. To create patient derived xenografts (PDXs) [2], mechanically minced patient PDAC tumor specimens were implanted subcutaneously into flanks of the mice, designated as passage 0 (P0). Expanded PDX tumors were excised and implanted into additional mice for expansion, designated P1 and subsequent passages as P(N+1). PDXs continued to be passaged and maintained in this manner until sufficient biological material was obtained for drug sensitivity testing. For this study, P3 PDX tumors were utilized. Tumor dimensions were measured by a digital caliper and estimated tumor volumes were calculated using the formula V= ½ (length × width^2). When tumors reached an estimated volume of 100 mm^3, mice were randomly divided into the following un-blinded treatment

groups (N=6 per group, N=24 total): vehicle control, TIV (150 mg/kg, oral gavage), PEM (30 mg/kg, IP injection twice weekly), TIV + PEM. These concentrations were utilized based on established human-to-murine conversion models [15]. No mice were excluded from this study for failure to reach estimated tumor volume. Study numbers were established *a priori.* Vehicle control and TIV were formulated in PEG 400 + 20% Vitamin E tocopheryl polyethylene glycol succinate (TPGS) (60:40) at 30 mg/mL, and orally administered for 5 consecutive days, followed by a 2 day dosing holiday, for four cycles. PEM was diluted in normal saline (0.9% NaCl). Body weight and tumor size were measured twice weekly over the 4-week study period. Relative tumor volumes were averaged and compared between treatment groups, where the relative tumor volume equaled the ratio of tumor volume at different time points to the initial tumor volume. Results were graphed using GraphPad Prism software.

IHC staining

Slides were sectioned at 4 µm and mounted on positively charged slides before baking at 58°C for a minimum of 1 h. H&E was performed per clinical protocols. Slide staining was carried out on a Ventana Discovery Ultra Autostainer. Antigen retrieval was carried out onboard with Ventana Cell Conditioning Buffer 1 (CC1) under standard conditions. Primary antibodies against ki-67 (1:100) and cleaved caspase-3 (1:150) were incubated at 37°C for 1 h followed by detection with Ventana OmniMap anti-rabbit-HRP (Roche, 7604311) and ChromoMap DAB (Roche, 760159) following the manufacturer's recommendations.

Table S2. Descriptive statistics of PDX studies.

	No Treatment			TIV			PEM			TIV + PEM		
Days	Relative Mean	SD	N	Relative Mean	SD	N	Relative Mean	SD	N	Relative Mean	SD	N
0	1.00	0.00	6	1.00	0.00	6	1.00	0.00	6	1.00	0.00	6
3	2.62	0.91	6	1.95	0.20	6	2.90	0.82	6	2.57	0.42	6
6	7.31	2.39	6	3.90	0.59	6	5.28	1.52	6	4.40	1.28	6
10	9.42	2.55	6	5.56	0.86	6	5.54	1.21	6	5.03	1.62	6

14	11.82	2.50	6	6.87	1.57	6	6.55	1.47	6	5.35	1.75	6
18	13.42	2.40	6	7.93	1.30	6	8.14	2.05	6	5.29	1.26	6
21	16.66	2.86	6	9.25	2.19	6	10.24	2.79	6	6.08	1.29	6
24	17.56	3.17	6	10.06	3.06	6	10.87	3.13	6	6.40	1.21	6

The ARRIVE guidelines 2.0: author checklist

The ARRIVE Essential 10

These items are the basic minimum to include in a manuscript. Without this information, readers and reviewers cannot assess the reliability of the findings.

Item		Recommendation	Section/line number, or reason for not reporting
**Study design**	1	For each experiment, provide brief details of study design including:<br>a. The groups being compared, including control groups. If no control group has been used, the rationale should be stated.<br>b. The experimental unit (e.g. a single animal, litter, or cage of animals).	-Materials and Methods: "In vivo drug testing"<br>-Supplementary Information: "In vivo drug testing"
**Sample size**	2	a. Specify the exact number of experimental units allocated to each group, and the total number in each experiment. Also indicate the total number of animals used.<br>b. Explain how the sample size was decided. Provide details of any *a priori* sample size calculation, if done.	a) Materials and Methods: "In vivo drug testing"<br>a, b) Supplementary Information: "In vivo drug testing"
**Inclusion and exclusion criteria**	3	a. Describe any criteria used for including and excluding animals (or experimental units) during the experiment, and data points during the analysis. Specify if these criteria were established *a priori*. If no criteria were set, state this explicitly.<br>b. For each experimental group, report any animals, experimental units or data points not included in the analysis and explain why. If there were no exclusions, state so.<br>c. For each analysis, report the exact value of *n* in each experimental group.	a, b, c) Supplementary Information: "In vivo drug testing"<br>c) Materials and Methods: "In vivo drug testing"
**Randomisation**	4	a. State whether randomisation was used to allocate experimental units to control and treatment groups. If done, provide the method used to generate the randomisation sequence.<br>b. Describe the strategy used to minimise potential confounders such as the order of treatments and measurements, or animal/cage location. If confounders were not controlled, state this explicitly.	-Materials and Methods: "In vivo drug testing"<br>-Supplementary Information: "In vivo drug testing"
**Blinding**	5	Describe who was aware of the group allocation at the different stages of the experiment (during the allocation, the conduct of the experiment, the outcome assessment, and the data analysis).	-Materials and Methods: "In vivo drug testing" -Supplementary Information: "In vivo drug testing"
**Outcome measures**	6	a. Clearly define all outcome measures assessed (e.g. cell death, molecular markers, or behavioural changes).<br>b. For hypothesis-testing studies, specify the primary outcome measure, i.e. the outcome measure that was used to determine the sample size.	a, b) Results: "PD-1 and MET inhibition effectively slows tumor growth in PDXs."<br>b) Supplementary Information: "In vivo drug testing"
**Statistical methods**	7	a. Provide details of the statistical methods used for each analysis, including software used.<br>b. Describe any methods used to assess whether the data met the assumptions of the statistical approach, and what was done if the assumptions were not met.	a, b) Materials and Methods: "In vivo drug testing"<br>b) Supplementary Information: "In vivo drug testing"
**Experimental animals**	8	a. Provide species-appropriate details of the animals used, including species, strain and substrain, sex, age or developmental stage, and, if relevant, weight.<br>b. Provide further relevant information on the provenance of animals, health/immune status, genetic modification status, genotype, and any previous procedures.	Supplementary Information: "In vivo drug testing"
**Experimental procedures**	9	For each experimental group, including controls, describe the procedures in enough detail to allow others to replicate them, including:<br>a. What was done, how it was done and what was used.<br>b. When and how often.<br>c. Where (including detail of any acclimatisation periods).<br>d. Why (provide rationale for procedures).	Supplementary Information: "In vivo drug testing"
**Results**	10	For each experiment conducted, including independent replications, report:<br>a. Summary/descriptive statistics for each experimental group, with a measure of variability where applicable (e.g. mean and SD, or median and range).<br>b. If applicable, the effect size with a confidence interval.	a) Table S2<br>b) Figure 6

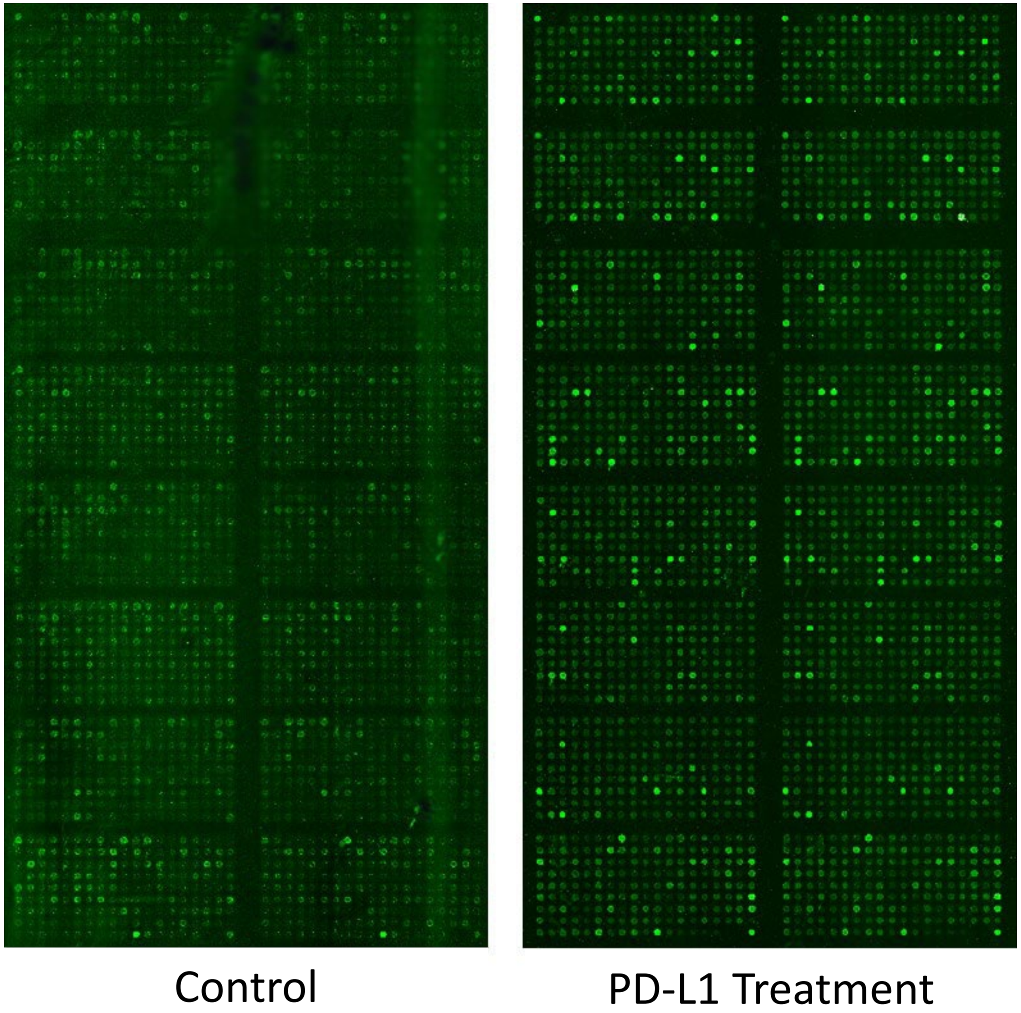

Figure S1. Phospho-protein Array Fluorescent Images of PANC-1 Cell Lysates After PD-L1 Treatment.

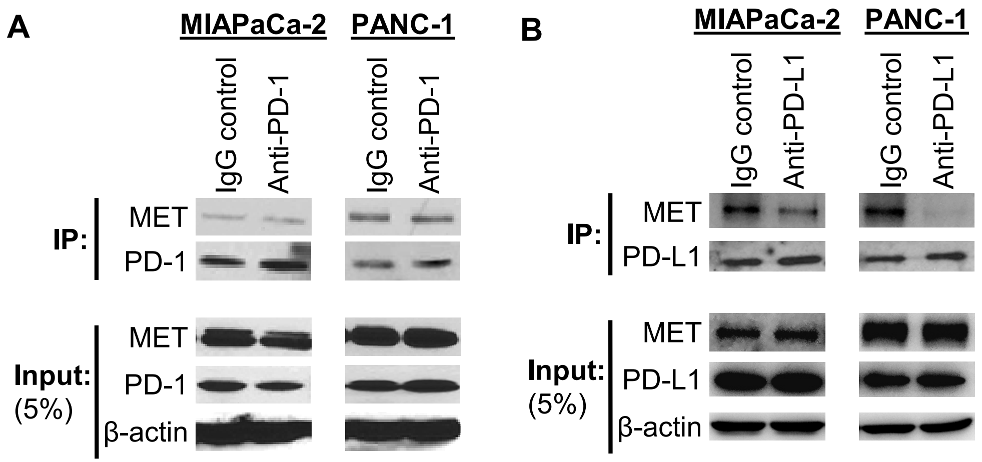

Figure S2. MET does not directly interact with PD-1 or PD-L1. Co-IP assays with PD-1 (**A**) and PD-L1 (**B**) pulldown. In the bottom panels, a 5% of whole cell lysates were immunoblotted and demonstrate innate MET, PD-1, and PD-L1 expression in PDAC cells. Whole lysates were then immunoprecipitated (IP) with IgG control, anti-PD-1 (**A**) , or anti-PD-L1 (**B**) antibodies. In the upper panels, IP pull-down lysates were immunoblotted with anti-MET, anti-PD-1, or anti-PD-L1 antibodies, respectively. MET expression was not enriched in anti-PD-1 or anti-PD-L1 vs. IgG control pull-downs in either cell line demonstrating that there is likely no direct interaction between the MET and PD-1 or MET and PD-L1.
