# Supplementary figures and images for: Endogenous Pancreatic Cancer Cell PD-1 Activates MET and Induces Epithelial-Mesenchymal Transition to Promote Cancer Progression
Source: Cancers (Basel). 2022 Jun 21;14(13):3051. doi: 10.3390/cancers14133051 (PMC9264908; doi:10.3390/cancers14133051)

**Fig. 1A**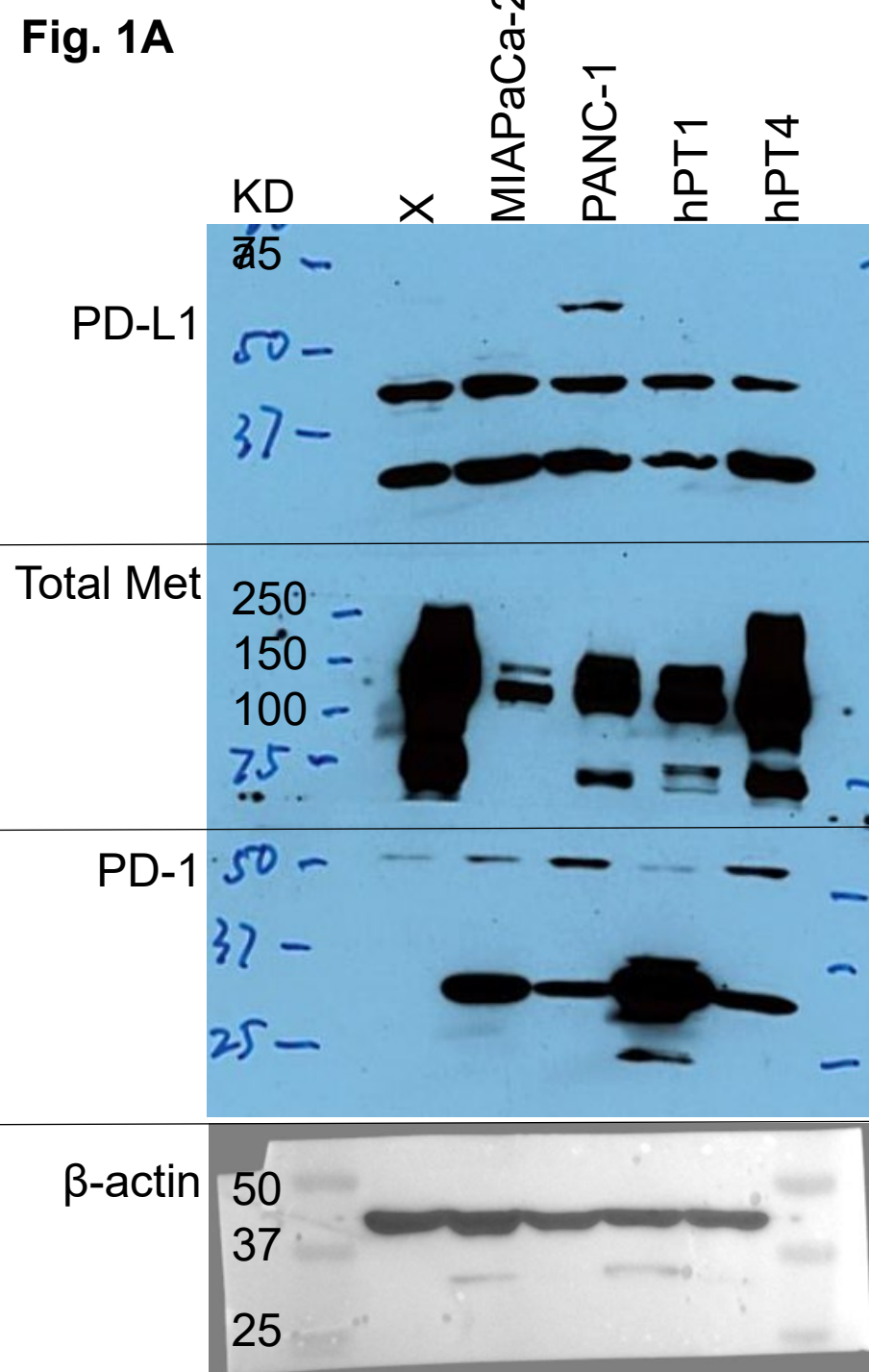**Fig. 1B**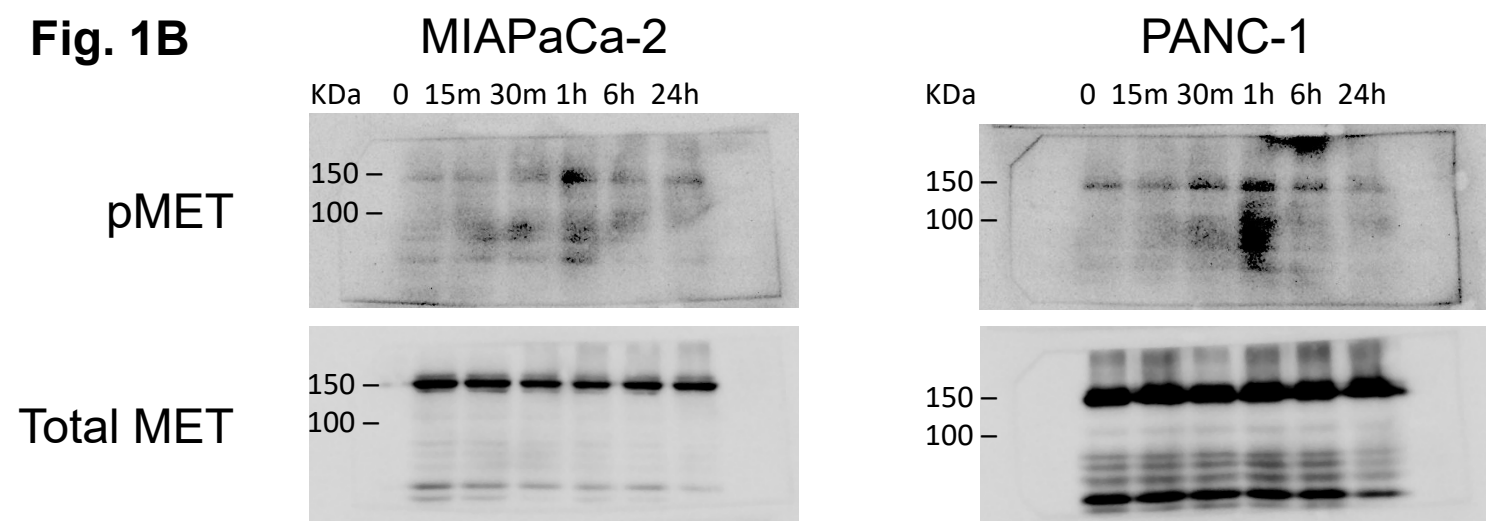**Fig. 1C**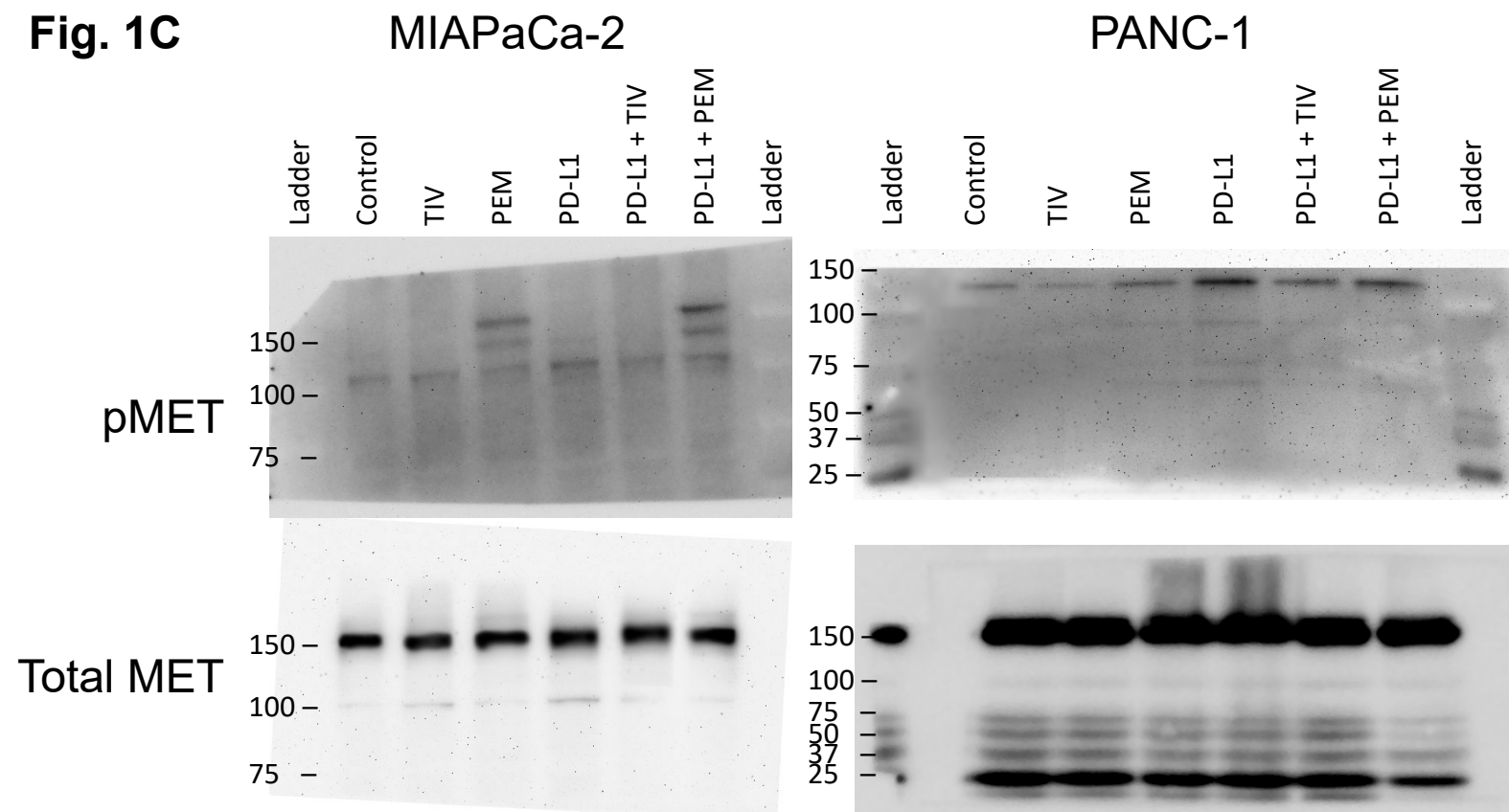

**Fig. 3A**

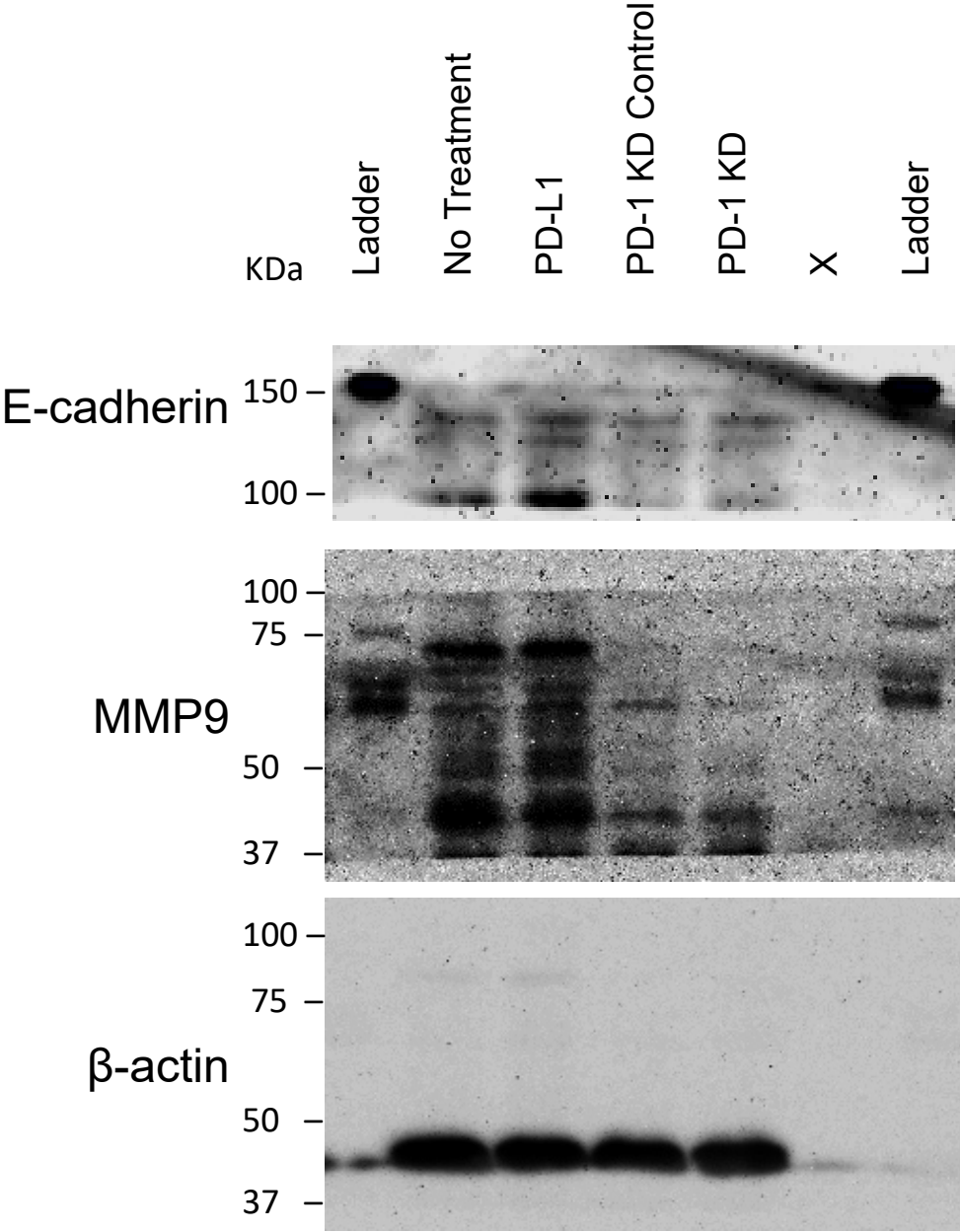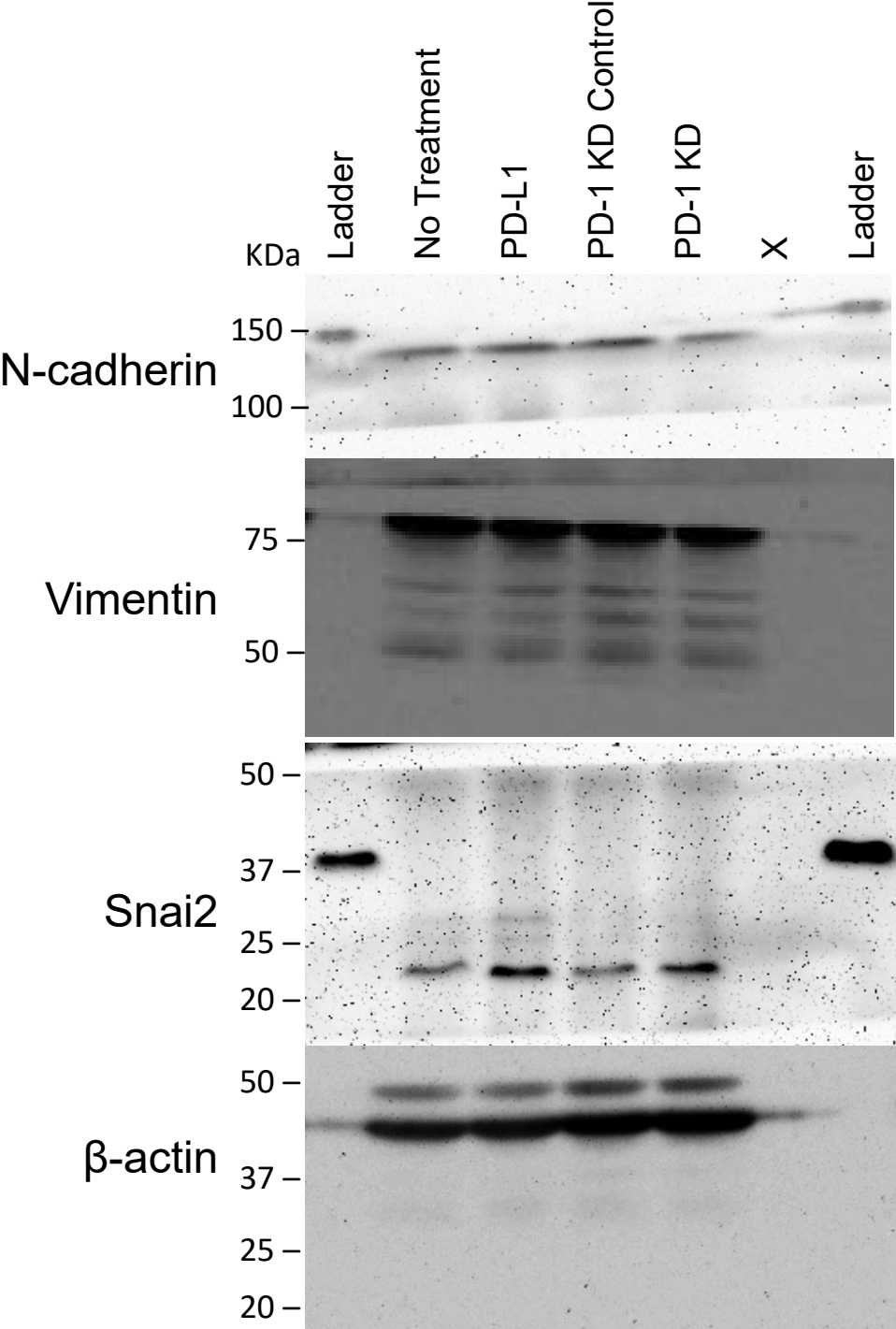

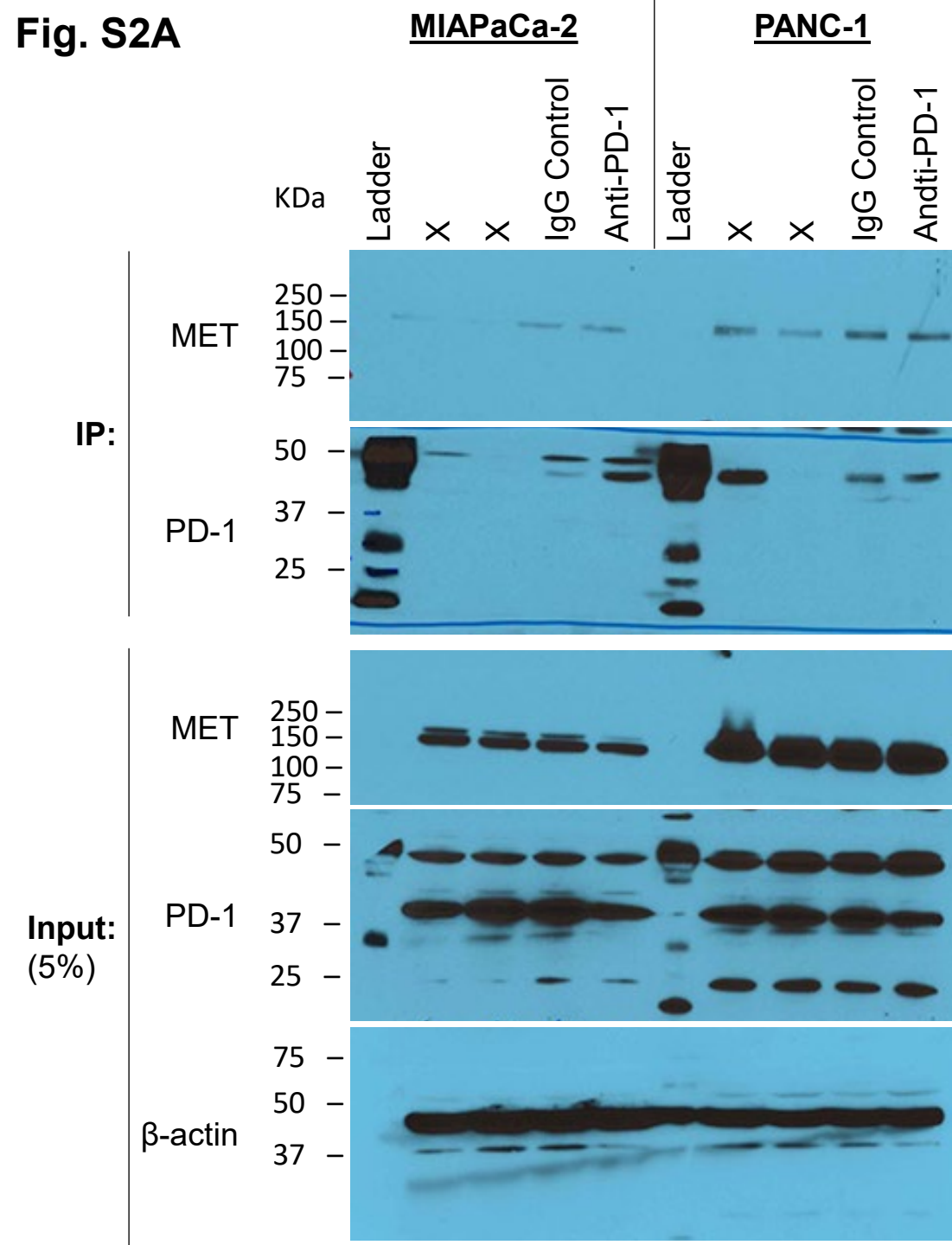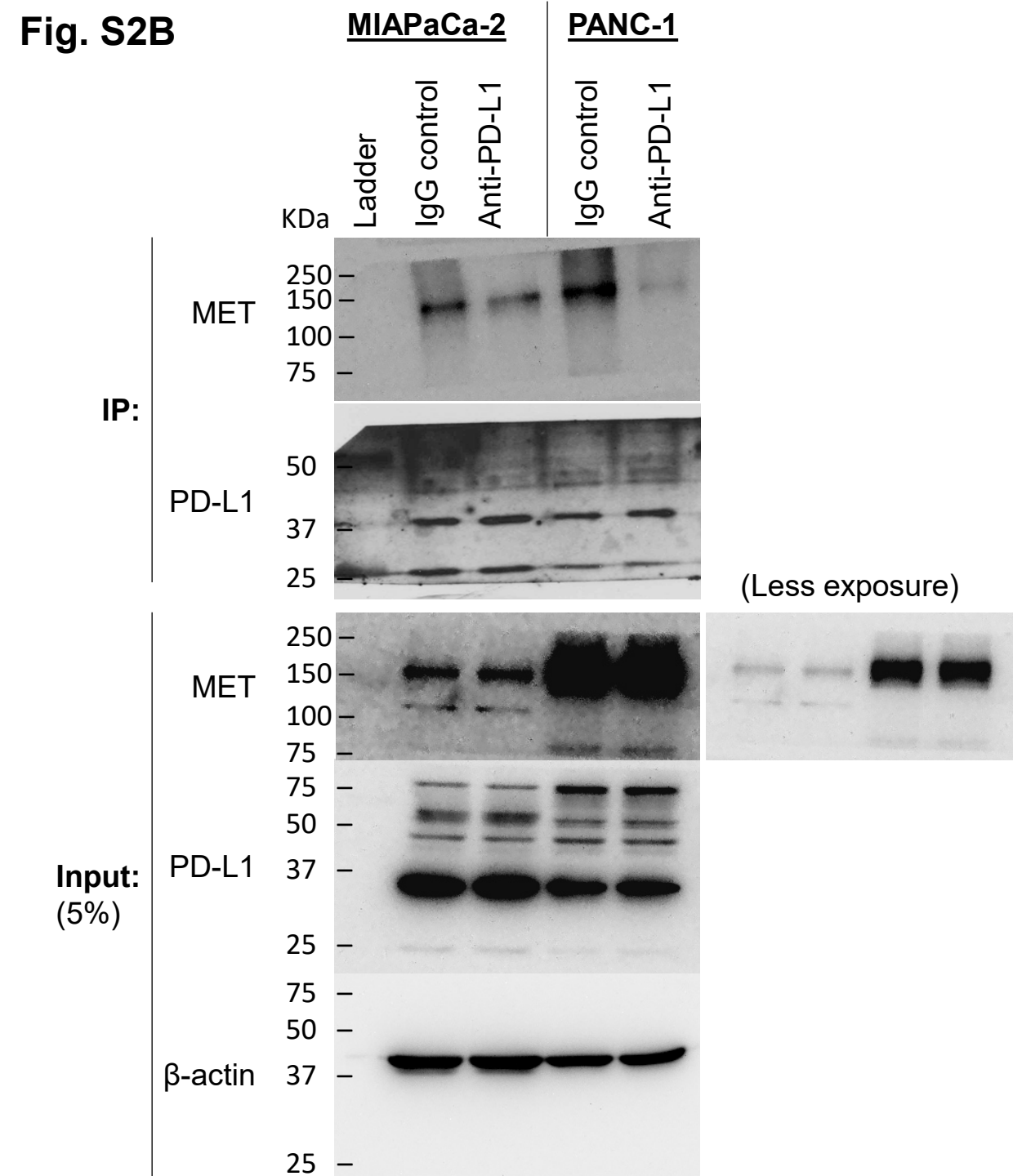

Supplement: Supplementary file 1 [file cancers-14-03051-s001.zip › Supplementary File S2.pdf]
